# Effects of *Nigella sativa, Camellia sinensis*, and *Allium sativum* as Food Additives on Metabolic Disorders, a Literature Review

**DOI:** 10.3389/fphar.2021.762182

**Published:** 2021-11-17

**Authors:** Akbar Anaeigoudari, Hamidreza Safari, Mohammad Reza Khazdair

**Affiliations:** ^1^ Department of Physiology, School of Medicine, Jiroft University of Medical Science, Jiroft, Iran; ^2^ Torbat Jam Faculty of Medical Sciences, Torbat Jam, Iran; ^3^ Cardiovascular Diseases Research Center, Birjand University of Medical Sciences, Birjand, Iran; ^4^ Applied Biomedical Research Center, Mashhad University of Medical Sciences, Mashhad, Iran

**Keywords:** metabolic disorders, *Nigella sativa* L., white tea, Garlic, anti-obesity effects, anti-diabetic effects, anti-inflammatory effects

## Abstract

**Objective:** Metabolic disorders (MD) can disturb intracellular metabolic processes. A metabolic disorder can be resulted from enzyme deficits or disturbances in function of various organs including the liver, kidneys, pancreas, cardiovascular system, and endocrine system. Some herbs were used traditionally for spices, food additives, dietary, and medicinal purposes. Medicinal plants possess biological active compounds that enhance human health. We aimed to provide evidence about therapeutic effects of some medicinal herbs on MD.

**Data Sources:** PubMed, Scopus, and Google Scholar were explored for publications linked to MD until February 2021. The most literature reports that were published in the last 10 years were used. All types of studies such as animal studies, clinical trials, and *in vitro* studies were included. The keywords included “Metabolic disorders,” “*Nigella sativa* L.,” “Thymoquinone,” “White tea”OR “*Camellia sinensis* L.” “catechin,” and “*Allium sativum* L.” OR “garlic” were searched.

**Results:** Based on the results of scientific studies, the considered medicinal plants and their active components in this review have been able to exert the beneficial therapeutic effects on obesity, diabetes mellitus and non-alcoholic fatty liver disease.

**Conclusions:** These effects are obvious by inhibition of lipid peroxidation, suppression of inflammatory reactions, adjustment of lipid profile, reduction of adipogenesis and regulation of blood glucose level.

## Highlights

*Nigella sativa* L., *Camellia sinensis* L., and *Allium sativum* L. as a food additive showed therapeutic effects on metabolic disorders.
These plants and their active components inhibited release of inflammatory mediators and oxidant parameters.
These plants and their active components showed antidiabetic, anti-inflammatory, antioxidant, lipolysis, hepatoprotective, and cardioprotective effects.

## Introduction

Metabolic diseases are disturbances that affect a wide range of intracellular metabolic processes (Bertrand and Lehuen, 2019). These diseases can be an outcome of enzyme deficits (Barbagallo and Dominguez, 2007) or perturbation in the function of vital organs/systems of the human body, including the liver, kidneys, pancreas, and cardiovascular system and endocrine system (Rochlani et al., 2017). In recent decades, the prevalence of metabolic diseases has been significantly enhanced, and it is considered as a serious threat for human health (Fan and Pedersen, 2020). One of the most common metabolic diseases affecting human health is type 2 diabetes mellitus (NCD Risk Factor Collaboration, 2016). Rapid prevalence of this disease among men and women mostly is attributed to obesity and increment of body mass index (BMI) (Esser et al., 2014). In addition, inflammation followed by overstimulation of the immune system has been recognized as an important player in pathogenesis of metabolic diseases such as type 2 diabetes mellitus (Rea et al., 2018). Accumulating evidence confirms that inflammation triggered by obesity has a basic contribution in the reduction of insulin secretion and insulin resistance (Boulangé et al., 2016). It has been also revealed that obesity caused by inflammation affects various organs such as the liver, heart, and brain and disturbs hemostatic mechanisms in the body (Saltiel and Olefsky, 2017). Scientific findings also demonstrate a link between increased levels of inflammatory markers such as C-reactive protein and type 2 diabetes mellitus (Yang et al., 2020). Besides inflammation, the role of oxidative stress in pathogenesis of metabolic degasses has been confirmed (Mostafavinia et al., 2021).

**FIGURE 1:**
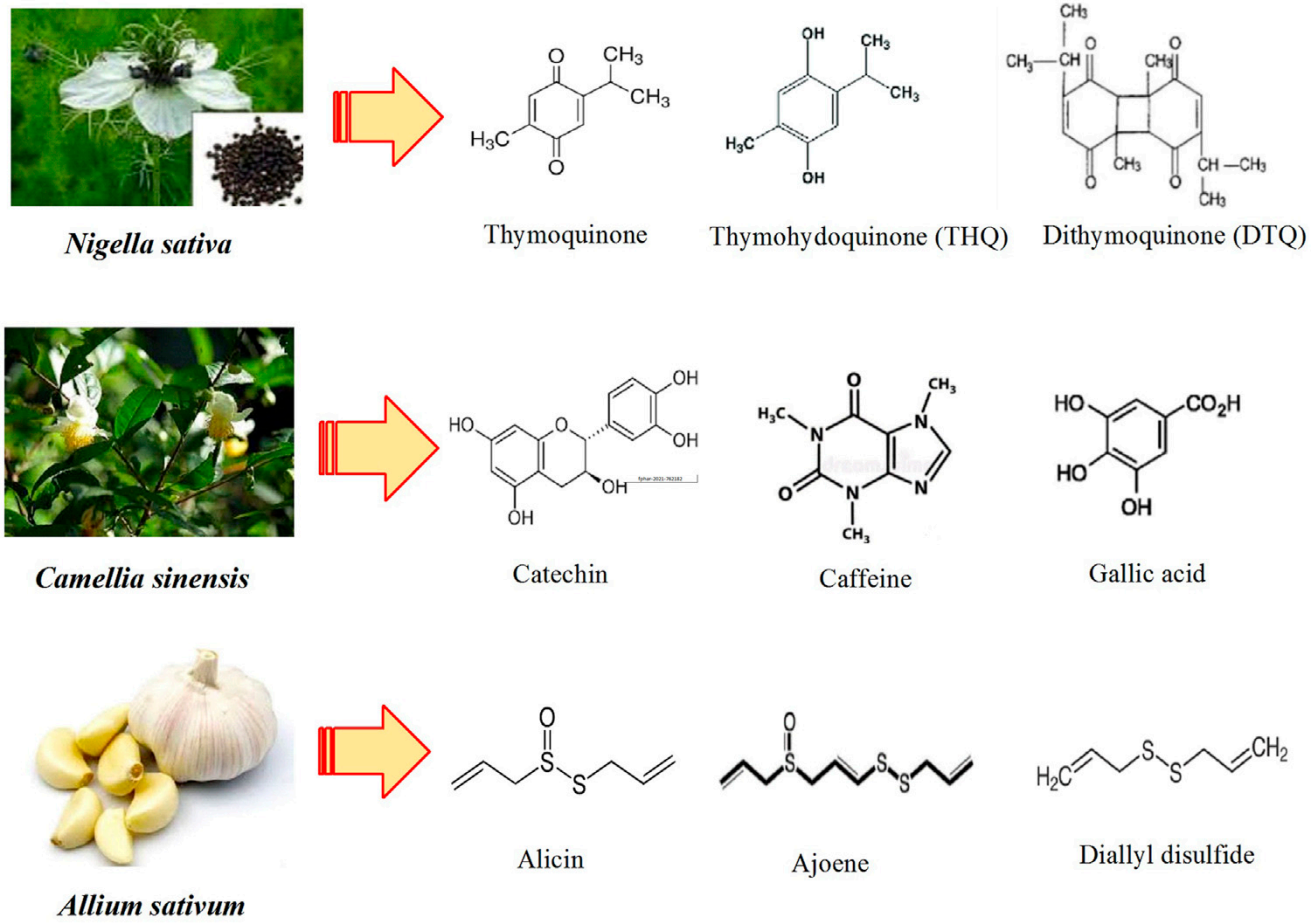
Chemical structures of main bioactive compounds of medicinal herbs.

Traditional medicine has been shown to have useful results in treatment of metabolic diseases (Zhang et al., 2020). Animal studies confirmed that extracts of some medicinal plants such as *Rhinacanthus nasutus* can modify overweight and lowered levels of glucose and lipids in rats (Ajmal Shah et al., 2017). Based on pharmacological research studies, flavonoids presented in the extract of plants such as *Scutellaria baicalensis* improved type 2 diabetes mellitus *via* attenuating inflammatory responses (Waisundara et al., 2008). It has been also documented that some varieties of tea also have potent effects in the improvement of nonalcoholic fatty liver disease (Mao et al., 2021). According to animal and clinical studies, plant alkaloids including berberine have also been suggested to have good therapeutic effects on obesity, type 2 diabetes mellitus, nonalcoholic fatty liver disease, and hyperlipidemia (Xu et al., 2020). *Nigella sativa* (*N. sativa*) and *Allium sativum* (*A. sativum*) are among the most commonly used medicinal plants in Persian traditional medicine (Khazdair et al., 2019). Traditionally, these plants have been used as dietary, food additives, spices, and various medicinal purposes for the treatment of different diseases (Gilani et al., 2004; Bathaie and Mousavi, 2010). Furthermore, *Camellia sinensis* is one of the most widely consumed functional beverages in the global population (Yi et al., 2019). Also, in this review article we documented the effects of some medicinal plants that were used as culinary and food additives on metabolic disorders. The selected medicinal plants and their active constituents are summarized in Figure 1.

## THE Effects of Medicinal Plants on Metabolic Disorders

### Nigella sativa

Black seed or *Nigella sativa* (*N. sativa*), a plant of the Ranunculaceae family, flourishes in different areas of the world and is used as food as well as a remedy in traditional medicine (Rusda et al., 2020). Thymoquinone (TQ), dithymoquinone (DTQ), thymol (THY), and thymohydroquinone (THQ) are main ingredients presented in the *N. sativa* extract (Figure 1). These compounds especially thymoquinone play an outstanding role in therapeutic effects of this plant (Randhawa and Al-Ghamdi, 2002). The oils of *N. sativa* are also rich in polyunsaturated fatty acids such as oleic, linoleic, and palmitic acids. In addition, carotenoids, sterols, tocols, and phenolics are present in oils of this plant (Ketenoglu et al., 2020).

**FIGURE 2:**
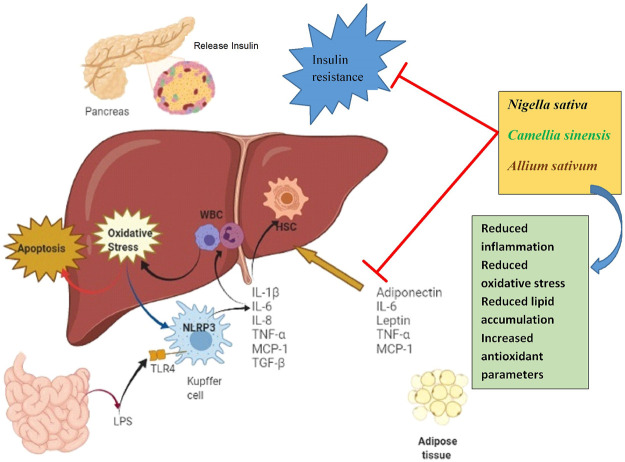
The potential therapeutic effects of three medicinal herbs on metabolic disorders.

A wide range of pharmacological effects such as modulation of immune system activity, suppression of inflammatory reactions, inhibition of oxidative stress, prevention of cancer cell proliferation, and death of germs have been attributed to *N. sativa* and its constituents (Adiban Fard et al., 2015; Mokhtari-Zaer et al., 2020). In addition, *N. sativa* has been demonstrated to cure disorders such as diabetes, dyslipidemia, and asthma (Namazi et al., 2018; Mortazavi Moghaddam et al., 2020). The effects of *N. sativa* in patients with metabolic syndrome illustrated that black seeds of *N. sativa* could improve body mass index (BMI), waist circumference, and fasting blood glucose. Co-administration of black seeds of *N. sativa* and turmeric also could ameliorate metabolic syndrome *via* the modification of BMI, waist circumference, fasting blood glucose, and lipid profile in patients (Ramadan, 2020). Anti-obesity of *N. sativa* and thymoquinone extracted from it has been also documented (Mahdavi et al., 2015). In addition, scientific evidence confirms that *N. sativa* regulates some of the membrane receptors involved in energy homeostasis including peroxisome proliferator–activated receptor gamma-2 (PPAR-γ2) (Benhaddou-Andaloussi et al., 2010). The potential molecular targets of the mentioned medicinal herbs on metabolic disorders are showed in Figure 2.

#### Effect of *N. sativa* and its Ingredient on Obesity

Obesity is a chronic metabolic disease which threatens human health. It can be a result of excessive accumulation of fat in adipocytes (Apovian, 2016). Obesity has been shown to affect the immune system for over-generation of pro-inflammatory cytokines including tumor necrosis factor alpha (TNF-α) (Ferrante, 2007). Obesity also can lead to the induction of nonalcoholic fatty liver disease. The beneficial effects of plant extracts in the improvement of MD have been documented. The researchers studied the effect of Livo-Pro-08 [*N. sativa* (250 mg/kg), *Entada pursaetha* (500 mg/kg) and *Ficus glomerata* (750 mg/kg)] on nonalcoholic fatty liver disease in rats. Based on results, the Liv-Pro-08 extract lowered the fasting blood glucose and insulin level. In addition, improvement of lipoprotein profiles and mitigation of liver enzymes such as alanine aminotransferase (ALT), aspartate aminotransferase (AST), and alkaline phosphatase (ALK-P) took place in rats treated by the extract (Vedanarayanan and Krishnan, 2011).

Thymoquinone (TQ) derived from the seeds and volatile oil of *N. sativa* has been suggested to be useful in blood glucose regulation and to ameliorate feeding disturbances (Mohtashami and Entezari, 2016). Uncoupling protein-1 (UCP-1) is an uncoupling molecule which disturbs the proton gradient in oxidative and phosphorylation in the mitochondria of brown adipose tissue (Dulloo et al., 2010). It has been understood that enhancing compounds of this protein are able to increase the metabolic energy consumption and reduce weight. In a study, the effect of TQ (100 mg/kg), hydroalcoholic (200 mg/kg), and hexane (300 mg/kg) extracts of *N. sativa* on the UCP-1 expression in brown adipose tissue was evaluated in mice. The results exhibited that TQ and both extracts augmented the expression of UCP-1. Based on results, TQ and extracts mitigated the level of cholesterol, low-density lipoprotein (LDL), and triglyceride (TG) and increased the concentration of high-density lipoprotein (HDL) in mice (Mahmoudi et al., 2018).

The ameliorative effect of TQ on the diabetic phenotype in a mice mold of diet-triggered obesity was investigated. In this study, the use of 20 mg/kg/day of this substance led to diminish the fasting blood glucose and insulin concentration and improve glucose tolerance and insulin sensitivity. In addition, the blood level of lipids including cholesterol and inflammatory mediators and liver TG decreased in animals treated by TQ. The fruitful effect of TQ on the diabetic phenotype was carried out through SIRT1-related pathways (Karandrea et al., 2017).

Peroxisome proliferator-activated receptors (PPARs), in particular PPAR-γ, have been reported to suppress the production of inflammatory cytokines (Motawi et al., 2017). It has been found that capsules of *N. sativa* (1,000 mg) could increase the expression of PPAR-γ and decreased TNF-α in obese women (Razmpoosh et al., 2019). In a clinical test, the effect of *N. sativa* (two capsules 750 mg) in obese men was checked. In this experimental test, the subjects received two capsules of *N. sativa* daily. The results determined that the body weight, fasting blood sugar, and cholesterol decreased while adiponectin increased in the group treated by *N. sativa* with respect to the untreated group (Datau et al., 2010).

#### Effect of *N. sativa* and its Ingredients on Diabetes Mellitus

Diabetes mellitus is a chronic and intricate metabolic disease which has affected a large number of people all over the world (Roglic et al., 2005). Type 2 diabetes mellitus as the most common form of diabetes can result from overweight as well as genetic aspects (Mellitus, 2006). *N. sativa* possesses antidiabetic properties (Yimer et al., 2019). *N. sativa* oil (400 mg/kg) has been reported to protect myocardial cells against streptozotocin-diabetic rats. This protective effect of *N. sativa* was mediated *via* inhibiting apoptosis and enhancing antiapoptotic factors such as Bcl-2 (Altun et al., 2019). Administration of 2 mg/kg of *N. sativa* oil also lowered fasting blood sugar and elevated the blood level of insulin in rats. In addition, antioxidant effects of *N. sativa* oil were mediated *via* the amplification of the activity of antioxidant enzymes including catalase and glutathione peroxidase (Abdelrazek et al., 2018). Combined extracts of *N. sativa* and *Cinnamomum cassia* (100 and 200 mg/kg) balanced the blood glucose and lipid profile in rats. It has been suggested that these two plants can be considered as an adjunctive therapy for diabetes (Kaur et al., 2018).

*N. sativa* oil (100 mg/kg) also could exert antidiabetic activities *via* mitigating the insulin/insulin receptor ratio and tumor necrosis factor-α in rats (Balbaa et al., 2016). Oral use of the ethanol extract of *N. sativa* (300 mg/kg) for 30 days considerably lowered the blood level of insulin, glucose, and lipids and attenuated lipid peroxidation and elevated antioxidant enzymes such as superoxide dismutase and catalase in the kidney and liver in diabetic rats (Kaleem et al., 2006). It has been documented that *N. sativa* and its essential oils alleviated hyperglycemia and ameliorated antioxidant capacity in diabetes mellitus caused by streptozotocin in rats (Sultan et al., 2014). *N. sativa* also could improve diabetic rabbits *via* a significant reduction in glucose and MDA concentrations as well as enhancement of GSH and ceruloplasmin levels (Meral et al., 2001). The fruitful effects of *N. sativa* on type 1 diabetes mellitus have been also documented. In a study, researchers reported that *N. sativa* oil (0.2 ml/kg) saved the islet of Langerhans against damages caused by streptozotocin in rats (Hmza et al., 2013). Researchers reported that administration of 50 and 100 mg/kg/day of TQ could reduce the level of glucose, HOMA-IR, and food and water intake in diabetic rats. These effects of TQ were carried out through enhancing the endogenous level of glucagon-like peptide 1 (Lee et al., 2019).

In a study, the impact of *N. sativa* (5.45 g/day) and fenugreek seed–supplemented chapatis in individuals with overweight and type 2 diabetes mellitus was appraised. In this study, BMI, fasting blood sugar, LDL, VLDL, and TG decreased in treated subjects (Rao et al., 2020). Administration of 2 g/day of *N. sativa* for 12 weeks has been shown to ameliorate dyslipidemia and to prevent the cardiovascular problem in patients with type 2 diabetes mellitus (Kaatabi et al., 2012).

In a clinical trial, the therapeutic effect of oral administration of *N. sativa* (2 g daily for 1 year) on type 2 diabetic patients was evaluated. Results identified that *N. sativa* administration decreased TG, total cholesterol, LDL, systolic blood pressure, diastolic blood pressure, mean arterial pressure, and heart rate (Badar et al., 2017).

Researchers examined the impact of *N. sativa* oil (2.5 ml, daily) on chronic kidney disease stages 3 and 4 resulted from diabetic nephropathy. Based on evidence, in patients treated by *N. sativa* oil the serum level of glucose, creatine, and urea was lower, and the glomerular filtration rate was higher when they were compared with that of the control group (Ansari et al., 2017).

#### Effect of *N. sativa* and its Ingredients on Nonalcoholic Fatty Liver Disease

Nonalcoholic fatty liver disease (NAFLD) is a metabolic disorder related to inflammation. Traditional medicine has been demonstrated to improve the liver dysfunction (Nishiyama et al., 2020). Researchers reported that a mixture of aqueous extracts including *N. sativa* seeds and fenugreek (100 mg/kg) has an improving effect on nonalcoholic fatty liver disease in rats. In addition, this mixture of aqueous extract could modify hyperglycemia *via* elevating the level of insulin and regenerating β cells in the islets of Langerhans (Mohamed et al., 2015).

TQ extracted from *N. sativa* has been shown to reverse NAFLD followed by hypothyroidism in rats. Based on this study, 400 mg/kg of TQ improved liver damage associated with hypothyroidism by over-expression of the antioxidant CAT gene (Ayuob et al., 2019). Administration of low dose (10 mg/kg) and high dose (20 mg/kg) of TQ improved oxidative stress *via* decreasing MDA and alleviated inflammatory responses through deccreasing the level of TNF-α and enhancing the IL-10 concentration in a rat model of NAFLD. In this study, the protective effect of high dose of TQ was more noticeable than that of the low dose (Awad et al., 2016). It has been determined that *N. sativa* (1 g twice a day) ameliorated liver function in NAFLD patients through lessening BMI, body weight, and liver enzymes (Hussain et al., 2017).

In a placebo-controlled clinical trial, 2 g/day of *N. sativa* seed could decrease the plasma level of inflammatory cytokines such as TNF-α, C-reactive protein, and nuclear factor kappa-B (NF-κB) in patients with NAFLD (Darand et al., 2019b). In another clinical trial, 1 g of *N. sativa* oil lowered the fasting blood sugar, TG, LDL, VLDL, liver enzymes, hs-C reactive protein, IL-6, and TNF-α levels in patients with NAFLD (Rashidmayvan et al., 2019). In a study, the effect of *N. sativa* seed oil (2.5 ml) on 60 patients with NAFLD was evaluated. The results indicated that *N. sativa* seed oil lessened the grade of hepatic steatosis, plasma level of aminotransferases, LDL-C, and TG and enhanced the content of HDL-C. There was not any significant difference in the weight of the group treated with oil compared to the placebo group (Khonche et al., 2019). It has been reported that tablets of Dava Al-Balgham prepared from a combination of *N. sativa* (105 mg), *Zataria multiflora* (105 mg), *Trachyspermum ammi* (105), and *Pistacia lentiscus* (105) reduced the liver enzymes level and weight in NAFLD patients (Hormati et al., 2019). Use of *N. sativa* (5 g) and *Melissa officinalis* (5 g) could lower the blood level of liver enzymes including AST and ALT, BMI, and degree fatty liver in patients with NAFLD in a clinical trial (Hosseini et al., 2018).

Based on the results of the studies, daily consumption of 2 g of *N. sativa* associated with lifestyle modification is useful in improvement of hepatic steatosis and insulin resistance in NAFLD patients (Darand et al., 2019a). The effect of 100 mg–20 g of seed powder, 20–80 mg of seed oil, 35–25 mg of TQ, and seed extract of *N. sativa* on reduction of the plasma level of lipids including cholesterol, LDL-C, and TG has been shown (Asgary et al., 2015). Effects of *N. sativa* and its ingredients on obesity, diabetes mellitus, and nonalcoholic fatty liver disease have been summarized in Table 1.

**TABLE 1:** Effects of *N. sativa* and its ingredients on obesity, diabetes mellitus, and nonalcoholic fatty liver disease.

Plant/ingredient	Effective dose	Type of study	Effects	Ref.
*N. sativa* extract	250 mg/kg	Rat	Reduced fasting serum level of glucose and insulin, improved lipoproteins profile, and mitigation of alanine aminotransferase, aspartate aminotransferase, and alkaline phosphatase	Vedanarayanan and Krishnan, (2011)
*N. sativa* and thymoquinone	200 and 300 mg/kg	Mice	Enhanced UCP-1, reduced cholesterol, LDL and TG, and increased HDL	(Mahmoudi et al., 2018)
	100 mg/kg			
Thymoquinone	20 mg/kg	Mice	Decreased fasting blood glucose and insulin concentration, cholesterol and inflammatory mediators and improved glucose tolerance and insulin sensitivity	Karandrea et al. (2017)
Capsules of *N. sativa*	1,000 mg	Clinical trial	Reduced the expression of PPAR-γ, adiponectin, and TNF-α in obese women	Razmpoosh et al. (2019)
capsules of *N. sativa*	1,500 mg	Clinical trial	Decreased body weight, fasting blood sugar, cholesterol as well as enhanced adiponectin	Datau et al. (2010)
*N. sativa* oil	400 mg/kg	Rat	Inhibited apoptosis and enhanced antiapoptotic factors such as Bcl-2	Altun et al. (2019)
*N. sativa* oil	2 mg/kg	Rat	Improved oxidative stress and enhanced antioxidant enzymes activities including catalase and glutathione peroxidase	Abdelrazek et al. (2018)
*N. sativa*	100 and 200 mg/kg	Rat	Regulated blood glucose and lipid profile	Kaur et al. (2018)
*N. sativa* oil	100 mg/kg	Rat	Improved diabetes *via* mitigating the insulin/insulin receptor ratio and tumor necrosis factor-α	Balbaa et al. (2016)
*N. sativa*	300 mg/kg	Rat	Decremented the blood level of insulin, glucose, lipids and attenuated lipid peroxidation and elevated antioxidant enzymes such as superoxide dismutase and catalase in the kidney and liver	Kaleem et al. (2006)
*N. sativa*	0.2 ml/kg	Rat	Protected the islet of Langerhans against damages caused by streptozotocin	Hmza et al. (2013)
TQ	50 and 100 mg/kg	Rat	Reduced glucose, HOMA-IR, food and water intake *via* enhancing the endogenous level of glucagon-like peptide 1	Lee et al. (2019)
*N. sativa*	5.45 g	Clinical trial	Decreased body mass index, fasting blood sugar, LDL, VLDL, and triglyceride	Rao et al. (2020)
*N. sativa*	2 g/daly	Clinical trial	Modified dyslipidemia and improvement of atherosclerosis and cardiovascular problems	Kaatabi et al. (2012)
*N. sativa*	2 g/daily	Clinical trial	Decreased triglyceride, total cholesterol, LDL, systolic blood pressure, diastolic blood pressure, mean arterial pressure, and heart (rate)	Badar et al. (2017)
*N. sativa* oil	2.5 ml daily	Clinical trial	Reduced glucose, creatine, urea, and higher glomerular filtration	Ansari et al. (2017)
*N. sativa* seeds and fenugreek	100 mg/kg	Rat	Improved the effect on nonalcoholic fatty liver, modification of hyperglycemia *via* elevating the level of insulin and regenerating of β cells in the islets of Langerhans of pancreas	Mohamed et al. (2015)
TQ	400 mg/kg	Animal (Rat)	Improved liver damage associated with hypothyroidism by over-expression of the antioxidant CAT gene	Ayuob et al. (2019)
TQ	10 and 20 mg/kg	Animal (Rat)	Improved oxidative stress *via* decreasing MDA and alleviated inflammatory responses through decrementing the level of TNF-α and enhancing IL-10	Awad et al. (2016)
*N. sativa*	1 g twice a day	Clinical trial	Decrease of BMI, body weight, and liver enzymes	Hussain et al. (2017)
*N. sativa*	2 g/day	Clinical trial	Decreased the plasma level of inflammatory cytokines such as TNF-α and decremented C-reactive protein and nuclear factor kappa-B (NF-κB)	Darand et al. (2019b)
*N. sativa* oil	1 g	Clinical trial	Decreased fasting blood sugar, TG, LDL, VLDL, liver enzymes including AST and ALT, hs-C reactive protein, IL-6, and TNF-α	Rashidmayvan et al. (2019)
*N. sativa* oil	2.5 ml	Clinical trial	Decreased grade of hepatic steatosis, plasma level of aminotransferases, LDL-C, and triglycerides and enhanced the content of HDL-C.	Khonche et al. (2019)
*N. sativa*	105 mg/kg	Clinical trial	Reduced liver enzymes level and weight in NAFLD patients	Hormati et al. (2019)
*N. sativa*	5 mg/kg	Clinical trial	Decreased blood level of liver enzymes including AST and ALT, BMI and degree fatty liver	Hosseini et al. (2018)
*N. sativa*	2 g	Clinical trial	Improved hepatic steatosis and insulin resistance	Darand et al. (2019a)
*N. sativa* oil and TQ	100 mg–20 g	Clinical trial	Reduced plasma level of lipids including cholesterol, LDL-C, and TG	Asgary et al. (2015)
	20–80 mg			
	35–25 mg			

### White Tea (*Camellia sinensis* L.)

White tea and other tea (green, oolong, and black) come from *Camellia sinensis*, or the tea plant. White tea prepared from young tea leaves is harvested only once a year in the spring (Rusak et al., 2008). The young tea leaves may be shielded from sunlight during growth for reducing chlorophyll formation and preparing white tea (Alcazar et al., 2007). The wide range of physiological and pharmacological properties for white tea including anticancer, anti-inflammatory and antioxidant (Dias, 2013), antiatherosclerotic and antihypertensive (Hodgson et al., 2005), and hypolipidemic and hypocholesterolemic effects (Huang and Lin, 2012) were reported. It has been suggested that oolong tea and dark tea could improve alcoholic fatty liver disease in mice by regulation of gut microbiota (Li et al., 2021). White tea contains proteins, minerals, and amino acids. Gallic acid, caffeine, and catechins are the main constituents of white tea (Ning et al., 2016) (Figure 1).

#### Effect of White Tea and its Constituent on Obesity

It has been reported that tea (white, green, and black) inhibited the pancreatic lipase activity. White tea was more effective than the other types. White and green teas have essentially equal amounts of flavan-3-ols. Green tea has high levels of flavonols. White tea also has high levels of 5-galloyl quinic acid, strictinin, trigalloyl glucose, and digalloyl glucose (Gondoin et al., 2010).

Incubation of preadipocytes with white tea extract (0.1, 0.25, 0.5 and 0.75%) significantly decreased TG during adipogenesis without affecting cell viability. Tea extract also showed lipolytic activity by increasing the content of free glycerol (32 μg/ml) in trated as compared to control cells. Tea extract downregulated the ADD1/SREBP-1c protein expression during adipogenesis and decreased SIRT1 mRNA levels compared to control cells (Söhle et al., 2009). These results indicated that white tea effectively inhibits adipogenesis and stimulates lipolysis activity. White tea extract (2.5%) and its main constituent catechins reduced the glucose and cholesterol uptake and enhanced LDL receptor binding activity and HDL concentration. Also, tea extract has been revealed to have a potent inhibiting capacity against lipase activity and TG levels in HepG2 cell lines (Tenore et al., 2013). The aqueous extract of white tea (1.5%) for 30 days, significantly decreased serum levels of glucose, LDL, cholesterol, and TG, while increased levels of HDL compared to control diabetic rats (Amanzadeh et al., 2020).

The modulating effects of teas on the plasma bile acids (BAs) profile were investigated. The plasma levels of murocholic acid, glycocholic acid, taurochenodeoxycholic acid, glycodeoxycholic acid, tauromuricholic acid, tauroursodeoxycholic acid, taurodeoxycholic acid, and taurocholic acid were increased, whereas levels of isolithocholic acid and taurolithocholic acid were decreased after drinking white, green, and oolong tea compared with control (Sun et al., 2019). Teas altered the bile acids metabolism, and this change could be associated with the health benefit effects of teas. White tea extract (0.5%) in induced obesity, reduced blood TG in male mice fed with a high-fat diet. White tea extract also reduced oxidative stress in the liver and adipose tissue. Moreover, tea extract was not able to reduce the food intake and body weight in animals (Teixeira et al., 2012).

The effects of white tea polysaccharide and polyphenol (400 or 800 mg/kg) on rats fed with high-fat diet (for 6 weeks) were investigated. These components suppressed body weight increases and fat accumulation. Moreover, polyphenols and polysaccharides improved blood lipid and antioxidant index levels as well as the reduction of the serum leptin levels and gene expression levels of IL-6 and TNF-α. Polysaccharides and polyphenols also showed synergistic effects in the reduction of serum leptin levels (Xu et al., 2015). These results indicated that the polysaccharide combination with polyphenols might be a potential therapy against obesity.

Use of low or high fat supplemented fed diets (5 and 30% triglyceride, respectively) with catechin (0.1, 0.2, and 0.5%) for 1 month significantly reduced body weight, visceral, and liver fat accumulation as well as development of hyperinsulinemia and hyperleptinemia in mice. Furthermore, treatment with catechins significantly increased acyl-CoA oxidase and medium chain acyl-CoA dehydrogenase mRNA expression and β-oxidation activity in the liver (Murase et al., 2002).

Administration of catechins (118.5 and 483.0 mg/day) in healthy male subjects (27–47 years) for 12 weeks significantly decreased BMI, waist circumference, body fat ratio, abdominal fat, and total cholesterol, glucose and plasminogen activator inhibitor-1 (PAI-1) in the serum compared to the baseline (Hase et al., 2001).

Treatment of an overweight participant (*n* = 107) with a beverage containing (625 mg of catechins with 39 mg caffeine) induced loss of body weight compared with the control group (39 mg caffeine) for 12 weeks. Percentage changes in total abdominal fat, subcutaneous abdominal fat, and fasting serum TG were greater in the catechin group than those of the control group (Maki et al., 2009).

#### Effect of White Tea and its Constituent on Diabetes

Administration of aqueous extracts of white tea **(**0.5%) significantly increased the drink intake compared to the normal control or diabetic control rats. Tea extracts significantly decreased blood glucose concentration and improved glucose tolerance ability compared to that of the diabetic group. In addition, liver weight and liver glycogen were remarkably increased, while total cholesterol and LDL-cholesterol were significantly decreased in the white tea–treated group compared to that of the diabetic animals (Islam, 2011).

White tea consumption (1%) in prediabetic rats significantly increased mRNA and protein expression levels of glucose transporters (GLUT1 and GLUT3) in the heart tissue. Administration of white tea also significantly increased cardiac acetate and alanine contents, antioxidant power of ferric, and expression and activity of lactate dehydrogenase (LDH) (Alves et al., 2015). Daily administration of white tea (1%) ameliorated glucose tolerance and insulin sensitivity. Also, tea extract decreased the protein expression levels of GLUT 1 and GLUT3 in the cortex of prediabetic rats. In addition, tea extract increased antioxidant capacity and suppressed lipid peroxidation and protein oxidation in the cortex of prediabetic animals (Nunes et al., 2015).

Treatment of diabetic rats with white tea extract (2%) significantly increased glutathione peroxidase (GSH-px), superoxide dismutase (SOD), and catalase (CAT) activities in the serum and liver compared to the diabetic control group (Al-Shiekh et al., 2014). The effect of white tea ethanolic extract (WTE) on the reduction of fasting blood glucose levels in diabetic rats showed that administration of WTE (100 mg/kg, BW) for 14 days decreased fasting blood glucose levels in diabetic rats. These effects can be attributed to the presence of flavonoid compounds such as catechins in tea extract (Ardiana et al., 2018). The use of catechins (25 and 50 mg/kg) modified the body weight of diabetic rats. Catechins also significantly reduced heart hypertrophy, plasma glucose levels, and matrix metallopeptidase 9 (MMP-9) levels. Moreover, catechin improved oxidative stress parameters in the nerves (Addepalli and Suryavanshi, 2018). Administration of (+)-catechin (0.2, 1.0, and 5.0 mmol/L) for 16 weeks significantly ameliorated renal dysfunction in type 2 diabetic mice and exerted protective effects against structural nephropathies. Also, catechin downregulated the level of NF-κB p65 phosphorylation and lowered pro-inflammatory mediators such as TNF- α and IL-1β in diabetic mice (Zhu et al., 2014).

Treatment of type 2 diabetes patients with catechin-rich beverages (96.3 mg) per day for 12 weeks in a double-blind controlled trial significantly decreased waist circumference compared to the control group. Additionally, catechin significantly increased adiponectin and insulin levels compared to the control group (Nagao et al., 2009).

Consumption of beverages containing catechin (540–588 mg) for 12 weeks significantly reduced abdominal fat, visceral fat area, subcutaneous fat area, body weight, and waist circumference and improved blood pressure in six human trials (*n* = 921, 505 men) (Hibi et al., 2018). These results indicated that white tea and catechin can reduce the risk of metabolic syndrome (MetS) due to the reduction of abdominal fat. The effects of beverages containing catechin (615 mg/350 ml) per day for 4 weeks on postprandial hyperglycaemia and oxidative stress in healthy postmenopausal women lowered postprandial glucose levels (3%) and serum postprandial thioredoxin (5%) compared to the placebo group. Catechins also increased antioxidant capacity and inhibited protein oxidation (Nunes et al., 2015).

#### Effect of White Tea and Its Constituent on Nonalcoholic Fatty Liver Disease

Long term consumption of white tea (15 mg/d or 45 mg/d) improved antioxidant activity and modified fatty acid profiles in a model of hepatotoxicity-induced Adriamycin (Espinosa et al., 2015).

White tea extract (200 μg/ml) significantly downregulated apolipoprotein B (APOB) and microsomal TG transfer protein (MTTP) expression and reduced the production of very-low-density lipoprotein (VLDL) in HepG2 cells. Tea extract stimulated LDL-cholesterol (LDL-c) uptake through activating the LDL receptor (LDLR). Furthermore, this extract significantly downregulated TG synthesizing enzyme genes and reduced intracellular TG accumulation (Luo et al., 2020). It has been reported that the catechins such as epigallocatechin-3-gallate (EGCG) and epicatechin-3-gallate (ECG) are abundant in white tea extract and contribute to the regulation of cholesterol metabolism (Luo et al., 2020).

Dietary supplements of white tea extract (5%) significantly reduced water intake and food consumption and lowered the serum total LDLc and TG levels. The tea extracts also significantly reduced lipid synthesis and blood glucose level, but increased glucose tolerance in mice. Furthermore, administration of the tea extract prevented the fatty liver formation and restored the normal hepatic structure (Teng et al., 2018). These results indicated that white tea has the protective effects on nonalcoholic fatty liver disease and metabolic disorders.

Co-administration of epigallocatechin-3-gallate (40–160 mg/kg) and caffeine presented in tea suppressed body weight gain and reduced white adipose tissue and energy intake than single use. These effects may be due to the alteration in the serum lipid profile, oxidative stress, and inflammatory cytokines in rats with NAFLD (Yang et al., 2019).

Histopathology results demonstrated that treatment of animals with NAFLD by (-) epigallocatechin gallate (EGCG) (85% pure extract) reduced number of fatty scores, necrosis, and inflammatory foci in liver tissue. EGCG also reduced liver injury and decreased fibrosis with downregulation in the expression of oxidative parameters and pro-inflammatory markers (Xiao et al., 2014).

Treatment of 17 patients with NAFLD (20–70 years) with low or high doses of catechins (1,080 mg/700 ml or 200 mg/700, beverage, respectively) or a placebo for 12 weeks in a double-blind randomized study reduced body fat. Moreover, the level of alanine ALT and urinary 8-isoprostane excretion significantly decreased in the high dose of catechin compared to the placebo and low dose groups after 12 weeks (Sakata et al., 2013).

The beneficial effects of catechin on hepatic dysfunction through the reduction of intracellular redox distress and inhibition of inflammatory reactions resulted from the nuclear factor kappa-B (NFκB) pathway activity have been reported (Hodges et al., 2020). In (Hodges et al., 2020). Effects of white tea and catechin on obesity, diabetes and nonalcoholic fatty liver disease are shown in Table 2.

**TABLE 2:** Effects of white tea and its ingredients on obesity, diabetes mellitus, and nonalcoholic fatty liver disease.

Plant/ingredient	Effective dose	Type of study	Effects	Ref.
White tea extract	0.1, 0.25, 0.5 and 0.75%	*In vitro* preadipocytes	Decreased TG incorporation during adipogenesis without effect on cell viability. Also increased lipolytic activity and downregulated ADD1/SREBP-1c protein expression during adipogenesis. It also decreased Sirt1 mRNA levels compared to control cells.	Söhle et al. (2009)
White tea extract	2.5%	HepG2 cell	Reduced the glucose and cholesterol uptake, while enhanced the LDL receptor binding activity and led to an increase in HDL cell medium concentration. Also, the tea extract revealed the best inhibition capacity against lipase activity and TG levels in cell lines.	Tenore et al. (2013)
White tea extract	1.5%	Rats	Decreased serum levels of glucose, LDL, cholesterol, and triglyceride, while increased levels of HDL compared to control diabetic rats	Amanzadeh et al. (2020)
White tea extract	0.5%	Mice	Reduced blood triacylglycerols associated with increased cecal lipids. White tea extract also reduced oxidative stress in the liver and adipose tissue. Moreover, tea extract was not able to reduce food intake and body weight in animals.	Teixeira et al. (2012)
White tea polysaccharide and polyphenol	400 or 800 mg kg^−1^	Rats	Suppressed body weight increases and fat accumulation. Moreover, polyphenols and polysaccharides improved blood lipid and antioxidant levels. In addition, reduced the serum leptin levels and gene expression levels of IL-6 and TNF-α. Furthermore, polysaccharides and polyphenols showed a synergistic effect in reduction of serum leptin levels and in anti-inflammatory activity.	Xu et al. (2015)
Catechin	0.1, 0.2 and 0.5%	Mice	Reduced body weight, visceral and liver fat accumulation as well as development of hyperinsulinemia and hyperleptinemia. Furthermore, treatment with catechins significantly increased acyl-CoA oxidase and medium chain acyl-CoA dehydrogenase mRNA expression and β-oxidation activity in the liver of mice.	Murase et al. (2002)
Catechins	118.5 and 483.0 mg/day	Clinical study	Significantly decreased weight, body mass index (BMI), waist circumference, body fat ratio, abdominal fat and total cholesterol, glucose and plasminogen activator inhibitor-1 (PAI-1) in the serum compared to the baseline in healthy male subjects.	Hase et al. (2001)
Beverage containing catechins	625 mg	Clinical study	Induced loss of body weight compared with the control group. Percentage changes in total abdominal fat, subcutaneous abdominal fat, and fasting serum TG were greater in the catechins compared with the control group.	Maki et al. (2009)
White tea extract	0.5%	Rats	Increased the drink intake compared to the normal control or diabetic control rats. Decreased blood glucose concentrations and improved glucose tolerance ability. Also, total cholesterol and LDL-cholesterol were significantly decreased.	Islam, (2011)
White tea extract	1%	Prediabetic rats	Increased mRNA and protein expression levels of glucose transporters (GLUT1 and GLUT3) in the heart tissue. White tea also increased cardiac acetate and alanine contents, Ferric reducing antioxidant power and lactate dehydrogenase (LDH) in protein expression and activity.	Alves et al. (2015)
White tea extract	1%	Prediabetic rats	Ameliorated glucose tolerance and insulin sensitivity. Decreased the protein expression levels of GLUT 1 and GLUT3 in the cortex of animals. Increased the antioxidant capacity and suppressed lipid peroxidation and protein oxidation in the cortex of prediabetic animals.	Nunes et al. (2015)
White tea extract	2%	Diabetic rats	Increased glutathione peroxidase (GSH-px), superoxide dismutase (SOD), and catalase (CAT) activities in the serum and liver compared to the diabetic control group.	Al-Shiekh et al. (2014)
White tea ethanolic extract	100 mg/kg, BW	Diabetic rats	Decreased fasting FBG level.	Ardiana et al. (2018)
Catechin	25 and 50 mg/kg	Diabetic rats	Decreased body weight. Reduced heart hypertrophy, plasma glucose levels, and MMP-9 levels. Improved oxidative stress parameters in the nerves.	Addepalli and Suryavanshi, (2018)
(+)-catechin	0.2, 1.0, and 5.0 mmol/L	Mice	Ameliorated renal dysfunction in type 2 diabetic mice, and protective effects against structural nephropathies. Downregulated the level of NF-κB p65 phosphorylation as well as lowered pro-inflammatory mediators such as TNF- α and IL-1β in diabetic mice.	Zhu et al. (2014)
Catechin-rich beverage	96.3 mg	Clinical study	Decreased waist circumference compared to the control group. Increased adiponectin and insulin levels in type 2 diabetic patients compared to the control group	Nagao et al. (2009)
Catechin-rich beverage	540–588 mg	Clinical studies	Reduced abdominal fat, visceral fat area, subcutaneous fat area, body weight, and waist circumference as well as improved blood pressure.	Hibi et al. (2018)
Catechin-rich beverage	615 mg/350 ml	Clinical study	Reduce the risk of metabolic syndrome (MetS) due to reduction of abdominal fat. Lowered postprandial glucose levels and serum postprandial thioredoxin. Increases antioxidant capacity and inhibited protein oxidation in postprandial hyperglycaemia.	Nunes et al. (2015)
White tea extract	200 μg/ml	HepG2 cells	Downregulated apolipoprotein B (APOB) and microsomal TG transfer protein (MTTP) expression and reduced production of very-low-density lipoprotein (VLDL) in HepG2 cells. Stimulated LDL-cholesterol (LDL-c) uptake through its targeting receptor (LDLR). Downregulated TG synthetic genes and reduced intracellular TG accumulation.	Luo et al. (2020)
White tea extract	15 mg/d or 45 mg/d	Rats	Improved antioxidant activity and the fatty acid profiles of the liver and heart microsomes on Adriamycin-induced hepatotoxicity	Espinosa et al. (2015)
White tea extract	5%	Mice	Reduced water intake and food consumption and lowered the serum total LDLc and TG levels. Reduced lipid synthesis related to gene fatty acid synthase and blood glucose level, but increased glucose tolerance. Prevented the fatty liver formation and restored the normal hepatic structure.	Teng et al. (2018)
Epigallocatechin-3-gallate and caffeine	40–160 mg/kg	Rats	Reduced white adipose tissue and energy intake than single use. These effects may be due to the alteration in serum lipid profile, oxidative stress, and inflammatory cytokines in rats with NAFLD.	Yang et al. (2019)
(-) epigallocatechin gallate (EGCG)	85%	Rats	Improved hepatic histology including reduced number of fatty score, necrosis, and inflammatory foci. Reduced liver injury, decreased fibrosis with downregulation in the expressions of oxidative parameters and pro-inflammatory markers.	Xiao et al. (2014)
Catechin-rich beverage	1,080 mg/700 ml or 200 mg/700	Clinical studies	Reduced body fat and improved the liver-to-spleen computed tomography (CT) attenuation ratio. Decreased the level of alanine aminotransferase (ALT) and urinary 8-isoprostane.	Sakata et al. (2013)

### Garlic (*Allium sativum* L.)

Garlic (*Allium sativum* L.) belongs to the Amaryllidaceae family. Garlic is a bulbous and aromatic plant that is used as food and traditional remedy for various diseases worldwide (Bayan et al., 2014). This medicinal plant possesses bioactive ingredients with anti-inflammatory and antioxidant properties (Shang et al., 2019). The use of garlic has been recommended to aid respiration and digestion as well as treatment of leprosy and parasitic infection in ancient Chinese and Indian medicine (El-Saber Batiha et al., 2020). Bulbs of garlic possess the several phytochemicals including ajoenes, allicin (allyl 2-propenethiosulfinate or diallyl thiosulfinate), and sulfides [diallyl disulfide (DADS Al-Snafi, 2013] (Figure 1). Several biological and pharmacological activities for garlic and its related compounds including anticarcinogenic, antioxidant, antibacterial, antifungal, antidiabetic, anti-atherosclerotic, and antihypertensive renoprotective activities have been reported (Rahman and Lowe, 2006).

#### Effects of Garlic and Its Ingredients on Diabetes

Treatment of alloxan-diabetic rats by *A. sativum* decreased their blood glucose levels (Sheela et al., 1995). Liu et al. investigated the effects of garlic oil and diallyltrisulfide on glycemic control in STZ-diabetic rats. Diabetic rats received garlic oil and diallyltrisulfide for 3 weeks at a dose of 100 and 40 mg/kg body weight. The administration of garlic oil and diallyltrisulfide led to rise in basal insulin concentration and improved glucose tolerance. Both garlic oil and diallyltrisulfide ameliorated the increased level of blood glucose in diabetic rats through the increase of insulin secretion and enhancement of insulin sensitivity (Liu et al., 2005).

Albajali et al. assessed the effects of *A. sativa* (100 mg/kg/day) on glucose level, insulin concentration in streptozotocin-diabetic animals. Results showed a significant decrease in the serum level of glucose and a remarkable enhancement in blood insulin concentration (Osman et al., 2012). Garlic has been also revealed to decrease the TNF-α level and to increase the IL-10 production in alloxan-diabetic rats (Hashem et al., 2008). Hashem et al. examined the dose-dependent protective effect of garlic against streptozotocin-induced oxidative stress in hepatic and intestinal tissues. Findings showed that garlic administration (250 and 500 mg/kg) significantly normalized the blood glucose in diabetic rats (Kanth et al., 2008).

The effects of garlic oil and diallyl disulfide on blood glucose regulation and renal function showed that the administration of garlic oil (100 mg/kg) and DADS (40 or 80 mg/kg) until 16 weeks after the induction of diabetes stimulated insulin secretion in STZ-diabetic rats. Researchers suggested that the long-term treatment with garlic oil can improve glucose tolerance and renal function in diabetic rats (Liu et al., 2006). Short-term use of garlic in diabetic rabbits by alloxan also reduced the blood sugar levels as effectively as tolbutamide (Augusti, 1975).

Garlic ethanolic extract administration (0.1, 0.25 and 0.5 g/kg body wt.) in STZ-diabetic rats for 14 days significantly decreased the glucose, total cholesterol, triglycerides, urea, uric acid, creatinine, AST, and ALT levels and also increased serum concentration of insulin. Garlic extract has also been shown to have better antidiabetic effect than glibenclamide (Eidi et al., 2006). Musabayane et al. indicated that the garlic extract reduced blood glucose through increasing the insulin level. Also, the garlic extracts and metformin did not affect serum concentration of insulin in nondiabetic rats, while glibenclamide increased the blood level of insulin (Musabayane et al., 2006). The administration of aged garlic extract (AGE) (5 and 10 ml/kg, p.o.) significantly prevented adrenal hypertrophy, hyperglycemia, and elevated corticosterone levels in mice (Kasuga et al., 1999).

Mariee et al. studied the effects of garlic on diabetic nephropathy and oxidative stress induced by STZ in rats. The data revealed that fresh garlic homogenate reduced the serum level of glucose, total triglyceride, and total cholesterol and prevented STZ-induced diabetic nephropathy possibly through the inhibition of kidney oxidative damage and increase of nitric oxide bioavailability (Mariee et al., 2009). In another study, diabetic patients received 3 cloves of raw garlic (1 clove = 1.2 g) daily in the morning in fasting condition (12–14 h) for 30 days. The results showed that raw garlic has a potent antidiabetic effect (Mirunalini et al., 2011). The metabolic effects of time-released garlic powder tablets in patients with type 2 diabetes mellitus indicated that garlic reduced cardiovascular disease risks (Sobenin et al., 2008).

Also, many studies confirmed that the garlic as an adjunct can be useful for treatment of hyperlipidemia in patients with type 2 diabetes by inhibiting lipogenesis and promoting lipolysis (Afkhami-Ardekani et al., 2006). In a study, the effect of raw crushed garlic on metabolic syndrome was investigated. The patients were treated with 100 mg/kg of raw crushed garlic two times a day. Raw crushed garlic significantly reduced blood pressure, TG, and fast blood glucose compared to the control subjects (Choudhary et al., 2018).

#### Effects of Garlic and Its Ingredients on Nonalcoholic Fatty Liver Disease

The hypoglycemic, hypercholesterolemia, and hypotriglyceridaemic effects of garlic were studied in STZ-diabetic rats. Administration of garlic extract (500 mg/kg) for 7 weeks reduced the serum level of glucose, cholesterol, and TG levels. These results suggested that garlic could be effective in managing the side effects of diabetes (Thomson et al., 2007). In another study, the cardiovascular effects of aqueous garlic extracts (5–20 mg/kg) on normotensive and hypertensive rats decreased the mean arterial blood pressure (MAP), heart rate (HR), and systolic and diastolic blood pressure in hypertensive rats as well as in normotensive rats compared to the basal levels (Nwokocha et al., 2011). The effects of garlic on alloxan-induced diabetic male rabbits lowered serum glucose levels [38.88%] and serum cholesterol [57%], after 30 days of treatment compared to the control group. These results indicated that garlic could be a beneficial anti-hyperglycemic effect in alloxan-induced diabetic rabbits (Mahesar et al., 2010).

Marc et al. showed that aged garlic extract (AGE) had no significant effect on insulin resistance. They also revealed that treatment with AGE also had no significant effect on oxidative stress or inflammation (Atkin et al., 2016). Also, the *A. sativum* aqueous extract on heavy metal improved the lipid profile *via* the regulation of low-density lipoprotein-cholesterol (LDL-C), very-low-density lipoprotein-cholesterol (VLDL-C), and TG level (Gupta et al., 2008). The antihypertensive effect of garlic aqueous extract in the two–kidney–one–clip (2K-1C) Goldblatt model showed that the single dose of garlic (50 mg/kg) had a maximum antihypertensive effect 2–6 h after administration. They suggest that garlic does have an effective antihypertensive ability (Al-Qattan et al., 1999). Intraperitoneal administration of the *A. sativum* aqueous extract (500 mg/kg) for 3 weeks significantly increased serum antioxidant levels compared to the pretreatment levels in both diabetic and hypertensive rats. In addition, the *A. sativum* extract decreased serum glucose and systolic blood pressure in diabetic and hypertensive rats, respectively (Drobiova et al., 2011).

Chan Wok Sohn et al. examined the effect of high temperature/high pressure–processed garlic on plasma lipid profiles in rats. They suggested that high temperature/high pressure–processed garlic may be useful as a functional food to improve the lipid profile (Sohn et al., 2012). Black garlic extracts (0.5 and 1.5%) downregulated lipid and cholesterol metabolism in rats fed by a high fat diet. As a result, the blood levels of total lipids, triglyceride, and cholesterol were decreased (Ha et al., 2015). It has been reported that S-allyl cysteine sulphoxide (SACS), a sulfur-containing amino acid of garlic, has significant antidiabetic effects in alloxan-induced diabetic rats. Administration of allicin at a dose of 200 mg/kg decreased the concentration of blood glucose and ameliorated diabetic condition and increased liver and intestinal HMG CoA reductase activity and liver hexokinase activity (Sheela and Augusti, 1992).

The effects of garlic tablet (300 mg), containing (1.3% allicin) twice daily in patients with type 2 diabetes mellitus for 12 weeks significantly reduced total cholesterol, LDL-C, while significantly increased HDL cholesterol in patients treated with garlic compared to the placebo-treated group (Ashraf et al., 2004). In a study, the patients with type 2 diabetes mellitus received garlic tablets at doses of 300, 600, 900, 1,200, and 1,500 mg per day, respectively, for 24 weeks. Based on results, a significant reduction in FBS and HbA1c was observed when compared with placebo (Khan, 2011; Mirunalini et al., 2011; Aslani et al., 2016). Treatment of diabetic patients with three small cloves of raw garlic (1 clove = 1.2 g) daily in fasting condition for 30 days significantly reduced the blood glucose level, serum cholesterol, TG, and LDL while increased the HDL fraction. Furthermore, garlic significantly improved SOD, catalase (CAT) and glutathione peroxidase (GPx) in erythrocytes of diabetic patients compared to the control group (Khan, 2011; Mirunalini et al., 2011; Aslani et al., 2016).

Treatment of hyperlipidemic patients (*n* = 112) with garlic (20 g) daily with 1 tablespoon lemon juice significantly decreased the total cholesterol, LDL-cholesterol, and fibrinogen compared to the control groups (Khan, 2011; Mirunalini et al., 2011; Aslani et al., 2016). Aged garlic extract did not affect blood glucose but TG concentrations declined after 3 months of intervention. Aged garlic extract intake also reduced hydroperoxide as an indicator of oxidative stress (Ahmed et al., 2012). These findings suggest that dietary supplementation of garlic may be beneficial in reducing blood pressure and oxidative stress in hypertensive individuals. Effects of garlic and its ingredients on diabetes and nonalcoholic fatty liver disease are summarized in Table 3.

**TABLE 3:** Effects of garlic and its ingredients on obesity, diabetes mellitus, and nonalcoholic fatty liver disease.

Plant/ingredient	Effective dose	Type of study	Effects	Ref.
Garlic (*A. sativum* L.) sulfoxide amino acids	200 mg/kg	Rat	Decreased blood glucose level and ameliorate diabetes as well as rats treated with glibenclamide and insulin	Sheela et al. (1995)
*A. sativum* oil and diallyltrisulfide	100 and 40 mg/kg	Rat	Improved glycemic control in diabetic rats through increased insulin secretion and increased insulin sensitivity	Liu et al. (2005)
*A. sativum*	100 mg/kg	Rat	Improvement in histological appearance of pancreatic beta cells of diabetes mellitus (DM)	Osman et al. (2012)
*A. sativum*	250 and 500 mg/kg	Rat	Normalized the blood glucose in the diabetic rats	Kanth et al. (2008)
*A. sativum* oil and diallyl disulfide	Garlic oil (100 mg/kg) and DADS (80 mg/kg)	Rat	Improved oral glucose tolerance and renal function	Liu et al. (2006)
*A. sativum* ethanoic extract	0.1, 0.25 and 0.5 g/kg, bw	Rat	Decreased the glucose, total cholesterol, triglycerides, urea, uric acid, creatinine, AST, and ALT levels and also increased the serum concentration of insulin.	Eidi et al. (2006)
*A. sativum*	5 and 10 ml/kg	Rat	Prevented adrenal hypertrophy, hyperglycemia and elevated corticosterone level	Kasuga et al. (1999)
*A. sativum*	200 and 400 mg/kg	Rat	Reduced serum glucose, total triglyceride, and total cholesterol	Mariee et al. (2009)
*A. sativum*	3.6 g, daily	Clinical trial	Decreased the mean FBG and PPBG levels	Mirunalini et al. (2011)
Garlic powder tablets	300 mg twice a day	Clinical trial	Decreased the serum level of TG	Sobenin et al. (2008)
*A. sativum*	500 mg/kg	Rat	Reduced serum glucose, cholesterol, and TG levels	Thomson et al. (2007)
*A. sativum* aqueous extract	5–20 mg/kg	Rat	Decreased blood levels	Nwokocha et al. (2011)
*A. sativum* aqueous extract	1% solution/kg, b.wt	Rabbit	Lowered the serum glucose levels and serum cholesterol	Mahesar et al. (2010)
*A. sativum* aqueous extract	250 mg/kg, b.wt	Rat	Improved LDL-C, VLDL-C, and TG levels	Gupta et al. (2008)
*A. sativum* aqueous extract	50 mg/kg	Female rats	Showed a maximum antihypertensive effect	Al-Qattan et al. (1999)
*A. sativum* aqueous extract	500 mg/kg, IP	Rat	Increased the serum antioxidant level. Decreased serum glucose and systolic blood pressure	Drobiova et al. (2011)
Black garlic extract	0.5 and 1.5%	Rat	Down-regulated lipid and cholesterol metabolism. Decreased levels of total lipids, TG, and cholesterol	Ha et al. (2015)
Allicin	200 mg/kg	Rat	Decreased the concentration of blood glucose and ameliorated diabetic condition and also increased liver and intestinal HMG CoA reeducates activity and liver hexokinase activity	Sheela and Augusti, (1992)
Garlic tablet	300 mg (containing 1.3% allicin) twice daily	Clinical trial	Reduced total cholesterol and LDL– C and increased HDL	Ashraf et al. (2004)
Garlic tablet	300, 600, 900, 1,200, and 1,500 mg	Clinical trial	Reduced FBS and HbA1c	Khan, (2011); Mirunalini et al. (2011); Aslani et al. (2016)
Raw garlic	3.6 g	Clinical trial	Reduced blood glucose level, cholesterol, TG, and LDL. Increased the HDL fraction. Improved SOD, CAT, and GPx	Khan, (2011); Mirunalini et al. (2011); Aslani et al. (2016)
Garlic daily, plus 1 tablespoon lemon juice	20 g	Clinical trial	Decreased total cholesterol and LDL-cholesterol. Reduced systolic and diastolic blood pressure	Khan, (2011); Mirunalini et al. (2011); Aslani et al. (2016)
Aged garlic extract	3,000 mg	Clinical trial	Declined serum TG concentrations	Ahmed et al. (2012)

## Conclusion

Based on the results of animal and clinical trial studies, the mentioned medicinal herbs and their effective ingredients have beneficial therapeutic effects on metabolic diseases including obesity, diabetes mellitus, and fatty liver. These therapeutic properties are mainly associated with the modification of lipid profile, reduction of serum level of glucose, improvement of oxidative stress, and inhibition of inflammatory responses.
